# Effects of Mechanical and Chemical Pretreatments of Zirconia or Fiber Posts on Resin Cement Bonding

**DOI:** 10.1371/journal.pone.0129690

**Published:** 2015-06-11

**Authors:** Rui Li, Hui Zhou, Wei Wei, Chen Wang, Ying Chun Sun, Ping Gao

**Affiliations:** Department of Prosthodontics, Stomatological Hospital, Tianjin Medical University, Tianjin, China; University of North Carolina at Chapel Hill, UNITED STATES

## Abstract

The bonding strength between resin cement and posts is important for post and core restorations. An important method of improving the bonding strength is the use of various surface pretreatments of the post. In this study, the surfaces of zirconia (fiber) posts were treated by mechanical and/or chemical methods such as sandblasting and silanization. The bonding strength between the zirconia (fiber) post and the resin cement was measured by a push-out method after thermocycling based on the adhesion to Panavia F 2.0 resin cement. The zirconia and fiber posts exhibited different bonding strengths after sandblasting and/or silanization because of the different strengths and chemical structures. The zirconia post showed a high bonding strength of up to 17.1 MPa after a combined treatment of sandblasting and silanization because of the rough surface and covalent bonds at the interface. This effect was also enhanced by using 1,2-bis(trimethoxysilyl)ethane for the formation of a flexible layer at the interface. In contrast, a high bonding strength of 13.9 MPa was obtained for the fiber post treated by silane agents because the sandblasting treatment resulted in damage to the fiber post, as observed by scanning electron microscopy. The results indicated that the improvement in the bonding strength between the post and the resin cement could be controlled by different chemical and/or mechanical treatments. Enhanced bonding strength depended on covalent bonding and the surface roughness. A zirconia post with high bonding strength could potentially be used for the restoration of teeth in the future.

## Introduction

Zirconia and fiber posts with resin cement are widely used to restore endodontically treated teeth with all-ceramic crowns due to their high mechanical properties and good biocompatibility [1, 2]. However, the restoration of teeth usually fails in the long term because of post debonding from the root canal. The debonding commonly occurs at the interface between the post and resin cement because of a low bonding strength [3–5]. An important method for preventing debonding is by improving the retentive force between the post and root canal. This strength depends on the bonding strength at two interfaces, specifically, the root canal-resin cement interface and the resin cement-post interface [6]. Currently, the bonding strength between the resin cement and root canal can be improved by using an adhesive primer and dentin bonding agents. Thus, the post debonding is mainly attributed to the low bonding strength between the post and resin cement [7].

Surface pretreatment of the post is considered an important method to improve the bonding strength because the treatment might result in chemical bonds at the interface and imparts a rough surface to the post [8,9]. Several methods of chemical and mechanical surface pretreatment have been reported such as sandblasting and the use of various silane agents. First, abrasion of airborne particles (sandblasting) with aluminum oxide (Al_2_O_3_) particles is commonly used for mechanical surface treatment. This method removes the contamination layers on the post surface, leading to a rough surface for micro-mechanical interlocking and a large bonding interface area for the post [10–12]. Second, many chemicals, including silane coupling agents and organofunctional trialkoxysilane esters, are used to treat the post surface to form chemical bonds at the interface between the post and resin cement [13]. The bonding strength is potentially improved by forming covalent bonds at the interface between the post and resin cement because of co-polymerization with unreacted C = C bonds of the monomers of the resin cement. Recently, γ-methacryloxypropyltrimethoxysilane (γ-MPTS) is mainly used as a silane coupling agent in clinical dentistry [14] to enhance the adhesion between ceramics and resin cements. The hydrolysis of the methoxy groups can be accelerated by acid in the solution and thus favors the formation of covalent siloxane bonds (-Si-O-Si-) with the post [15]. In addition, it is well known that the silane agent used is also an excellent wetting agent, which effectively increases the bonding strength because it improves the intimacy of the surface contact to the cement [16]. This feature is because silanes usually dimerize to produce intermediate surface coatings rather than react to the surface. This effect depends on the availability of silanol groups on silica.

The surface treatment is also controlled by the chemical properties of the post. Zirconia is acid resistant and does not react to the silanation, which is different from silica-based porcelain crowns that react with silane coupling agents [17]. Previous studies reported that [18,19] the resin cement containing the phosphate ester monomer 10-methacryloyloxydecyl dihydrogen phosphate (MDP) reacts to zirconia to form chemical bonds at the interface. This reaction offers a great potential to improve the bonding strength between the zirconia post and resin cement. However, few studies have reported the high bonding strength [20,21] and good long-term reliability of zirconia posts treated by MDP. The related studies are limited by two reasons: 1) the bonding strength at the interface of the zirconia post and resin is lower than that between common ceramics and the resin after coupling, and 2) there is no change in the surface and hydrophobicity of zirconia [22]. This problem can be partially overcome by mechanical treatment. The airborne-particle abrasion might generate specific particles on the surface of the zirconia post, which might react with the silane coupling agent to form chemical bonds. Several studies [23–25] demonstrated that hydrochloric acid accelerated the hydrolysis of γ-MPTS, and the introduction of 1,2-bis(trimethoxysilyl)ethane (BTS) [26–29] in γ-MPTS as a mixed silane increased the bond durability of the resin on the ceramic surface. Thus, a combined effect of chemical and mechanical treatment is important to improve the bonding strength between the post and resin cement.

In this paper, mechanical and/or chemical treatment including sandblasting and silane agents were applied on fiber and zirconia posts. Different conditions were used to investigate the bonding performance and fracture mode. The bonding strength between the zirconia or fiber posts and the resin cement was measured using a push-out test. The increase in the bonding strengths of posts treated by sandblasting, silanization and the combined treatment was analyzed. The results indicated that the zirconia post showed the highest bonding strength after sandblasting and silanization using a two-bottle primer, whereas the silane agent effectively increased the bonding strength of the fiber post.

## Materials and Methods

**Fig 1:**
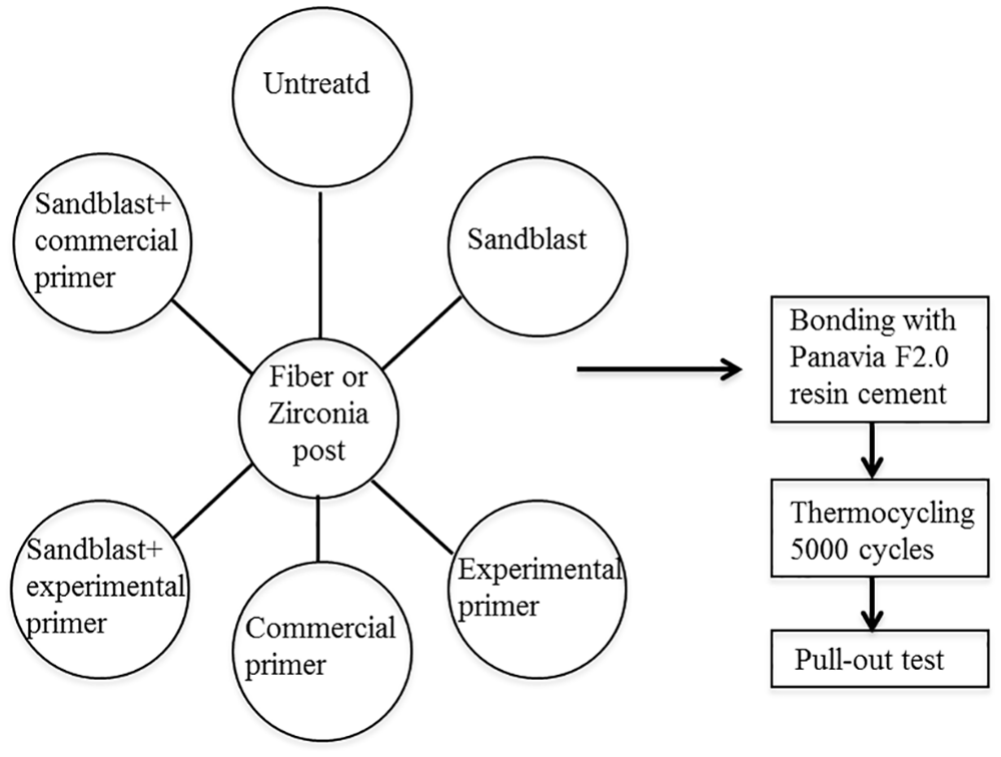
Schematic illustration of the measurement from the treatment.

Six surface treatment conditions including sandblasting and silane agents for two posts (fiber and zirconia) were used. The bonding strengths between the post and resin cement of the specimens were measured by a push-out test [30–33] after thermal cycling. A schematic illustration of the measurement from the treatment, thermocycling and push-out test is shown in Fig 1. These specimens were not aged. All of the conditions were statistically analyzed to determine the effects of the treatments on high bonding strength.

### Materials

The materials and their manufacturers are shown in Table 1. A commercially available fiber (diameter of 1.5 mm and length of 12 mm) was purchased from TENAX Company in the USA. Y-TZP zirconia bar (diameter of 1.5 mm and length of 12 mm) was purchased from the Lava Zirconia 3M ESPE Lab. γ-MPTS and BTS were purchased from ShinEtsu Chemical Industry and Tokyo Chemical Industry in Japan, respectively. A one-bottle silane primer (Monobond-s) was purchased from Ivoclar Vivadent Company. A polytetrafluoroethylene (PTFE) cylinder mold was purchased from ABC Company. All other chemicals were obtained from Beijing J&K Company and were used without further treatment.

**Table 1:** Materials and their manufactures in this study.

Materials	Composition	Manufacturer	Lot umber
Monobond-s	Ethanol: 50–100%, γ-methacryloxypropyle trimethoxy silane<2.5%	Ivoclar Vivadent, Liechtenstein	S05674
γ-MPTS	γ-methacryloxypropyletrimethoxy silane	ShinEtsu Chemical Industry, Tokyo, Japan	901770
BTS	1,2-Bis(trimethoxysilyl) ethane	Tokyo Chemical Industry, Tokyo, Japan	HH3SE
Panavia F 2.0	10-methacryloyloxy decyldihydrogenphosphate	Kuraray, Tokyo, Japan	071200

### Preparation of the experimental primer for two-bottle treatment

Two experimental primers were prepared in this study. Primer A was a silane solution, and primer B was an acid solution to accelerate the silane hydrolysis. Primer A was prepared by dissolving the silanes (50 mg) γ-MPTS and BTS in 1 mL ethanol with a molar ratio of 7:3. Primer B was prepared by the following process: Firstly, a hydrochloric acid (0.05 mol/L) solution was obtained by diluting a hydrochloric acid solution (0.1 mol/L, Wako Pure Chemical Industries, Osaka, Japan) with distilled and de-ionized water (pH = 6.0). The pH value of the hydrochloric acid solution (0.05 mol/L) was 1.66 as measured by a pH meter (Shanghai Rex PHS-3). Primer B was obtained after adding the above hydrochloric acid solution to ethanol with a volume ratio of 1:1.

### Surface treatment of the post

Fully sintered zirconia and fiber posts were treated by mechanical and/or chemical methods including sandblasting and silane coupling (one-bottle or two-bottle). The one-bottle treatment used commercial silane, and the two-bottle treatment was performed by mixing primers A and B. Six treatment conditions for the two posts (fiber: F-1, F-2, F-3, F-4, F-5 and F-6 and zirconia: Z-1, Z-2, Z-3, Z-4, Z-5 and Z-6) were used, as shown in Table 2.

**Table 2:** The surface treatment condition of the fiber and the zirconia posts.

Group		Treatment		
		sandblasting	silane	
			one-bottle	two-bottle
**Fiber Post**	F-1	---	---	---
	F-2	Yes	---	---
	F-3	---	Yes	---
	F-4	---	---	Yes
	F-5	Yes	Yes	---
	F-6	Yes	---	Yes
**Zirconia Post**	Z-1	---	---	---
	Z-2	Yes	---	---
	Z-3	---	Yes	---
	Z-4	---	---	Yes
	Z-5	Yes	Yes	---
	Z-6	Yes	---	Yes

F-1 and Z-1: No treatment was applied on the post.

F-2 and Z-2: The surface of the post was treated by airborne-particle abrasion with Al_2_O_3_ particles (diameter of 50 μm) using an oral Microblaster (Dento-prep, Ronviga, Daugaord, Denmark) for 10 seconds at 0.25 MPa from a distance of 10 mm. Then, the post was rinsed with 96% ethanol and dried in oil-free compressed air to remove the residual particles.

F-3 and Z-3: The surface of the post was treated with a commercial one-bottle type silane primer for 60 seconds, and then the post was dried in air for 10 seconds according to the manufacturer’s instruction.

F-4 and Z-4: The surface of the post was treated with the two-bottle type experimental primers, A and B. Typically, two single drops each from primer A and primer B were collected in a mixing dish and kept for 10 seconds. Thereafter, the mixed primer (0.3 mg) was applied onto the post for 1 min for the evaporation of ethanol at room temperature (23 ± 1°C). Then, the silanated post was dried for 2 min at room temperature.

F-5 and Z-5: The surface of the post was treated by sandblasting and the one-bottle type primer with the same conditions as F-2 (Z-2) and F-3 (Z-3), respectively.

F-6 and Z-6: The surface of post was treated by sandblasting and the two-bottle type primer with the same conditions as F-2 (Z-2) and F-4 (Z-4), respectively.

### Preparation of the specimens

After the surface treatment, the fiber or zirconia posts were placed vertically on a glass plate using sticky wax. Polytetrafluoroethylene (PTFE) cylinder molds (diameter of 6 mm and length of 12 mm) were set vertically around the post. The post was located in the center of the cylinder. A dual-curing resin cement, Panavia F 2.0 (Kuraray, Tokyo, Japan)[34], was then filled inside the circular hole of the cylinder mold.

Then, this adherent specimen was irradiated by visible light (Coltolux LED, Coltene, OH, USA) for 120 seconds. The power density of the light source was 450–600 mW/cm^2^, as determined by a light checker (3M Light Checker, 3M Health Care, Tokyo, Japan). The cylinder mold was carefully removed after the excess cement was wiped off. Each sample of resin cement/post was sectioned under water-cooling at a speed of 800 rpm and a fixed load of 200 g (Isomet 1000, Buehler, Illinois, USA) into 5 disc specimens (6 mm in diameter, 2 mm in thickness). These cut disc specimens were used for the push-out test.

After standing in water at 37°C for 1 day, the specimens were treated by thermocycling. The specimens were placed in a water bath between 5 and 55°C for 5,000 cycles for each bath (TC-501F, Weier, Suzhou, China). The dwell time in the water bath was 60 seconds. The transfer timer was 7 seconds.

### Measurement

#### Push-out test

**Fig 2:**
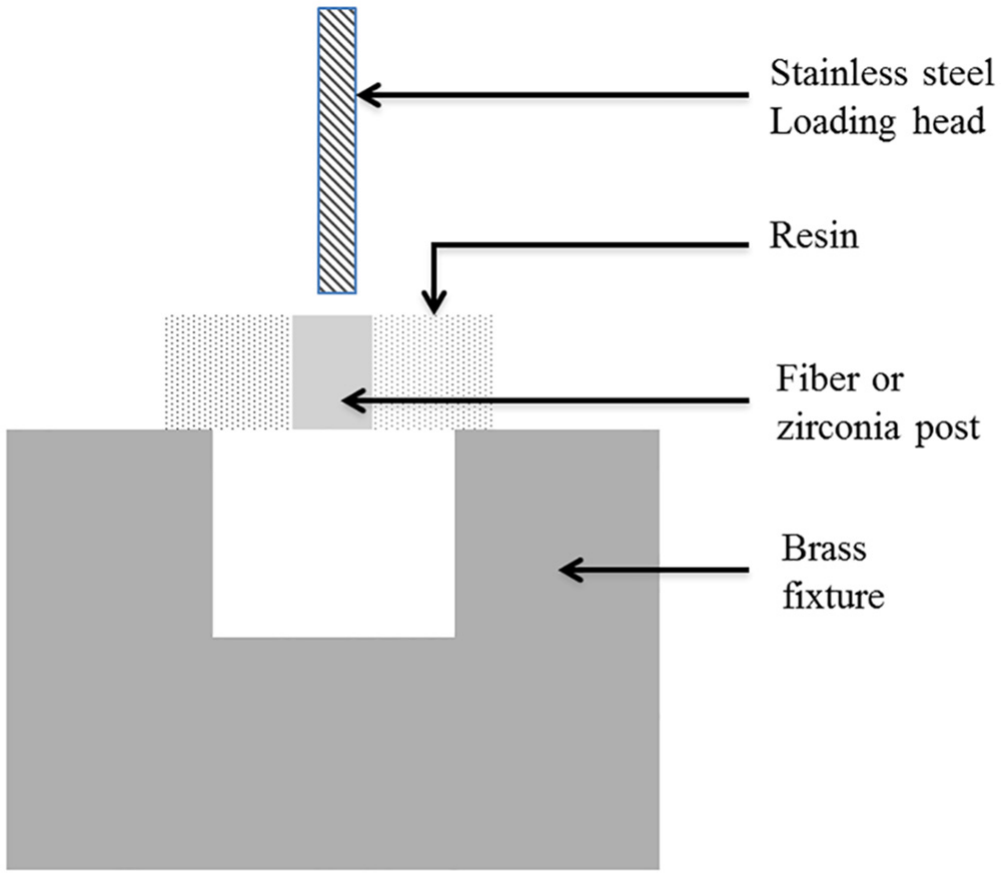
Schematic illustration of the push-out test.

Fig 2 shows a schematic illustration of the push-out test. The specimen was placed horizontally using double-sided adhesive tape on the copper carrier, which contained a 2-mm-diameter circular hole in the horizontal surface of the carrier. A stainless steel rod (1-mm diameter, 10 mm length) with a flat surface was held by a universal testing machine (Instron 3367, MA, USA), which was used as a loading head for pulling the post out of resin cement. The bonding strength was measured under a crosshead speed of 0.5 mm/min by the universal testing machine. The maximum load record in the push-out test indicated the degree of retention. The number of disc specimens for each experimental group was 15.

#### Fracture mode

After the push-out test, a digital microscope (MSV 330, Anyty, 3R, Japan) was used to observe the fracture. The fracture modes for all of the specimens were classified into three types: 1) cohesive failure of the resin cement or post, 2) mixed failure consisting of interfacial failure and cohesive failure, and 3) interfacial failure at the interface between the resin cement and post.

#### SEM observation

The surface morphology of the post before and after sandblasting and the bonding performance between the post to resin cement were observed by SEM (Hitachi S-4800). All of the samples were observed after gold coating.

### Statistical analysis

All of the data are expressed as the means ± standard deviations of a representative of similar experiments carried out in triplicate. Statistical analysis was performed by one-way analysis of variance (ANOVA) in conjunction with Tukey’s post hoc test for multiple comparisons using the SPSS (SPSS Software, San Diego, CA, USA) statistics program with a significance of P < 0.05. The differences were determined with Student’s t-test for each independent variable, and one-way analysis of variance (ANOVA) was conducted on all of the samples.

The fracture modes for all of the experimental groups were analyzed by the complex chi-square (χ^2^) test to determine significant differences in the three types of fracture modes. The statistical significance was set at the 0.05 level.

## Results and Discussion

### Morphology

**Fig 3:**
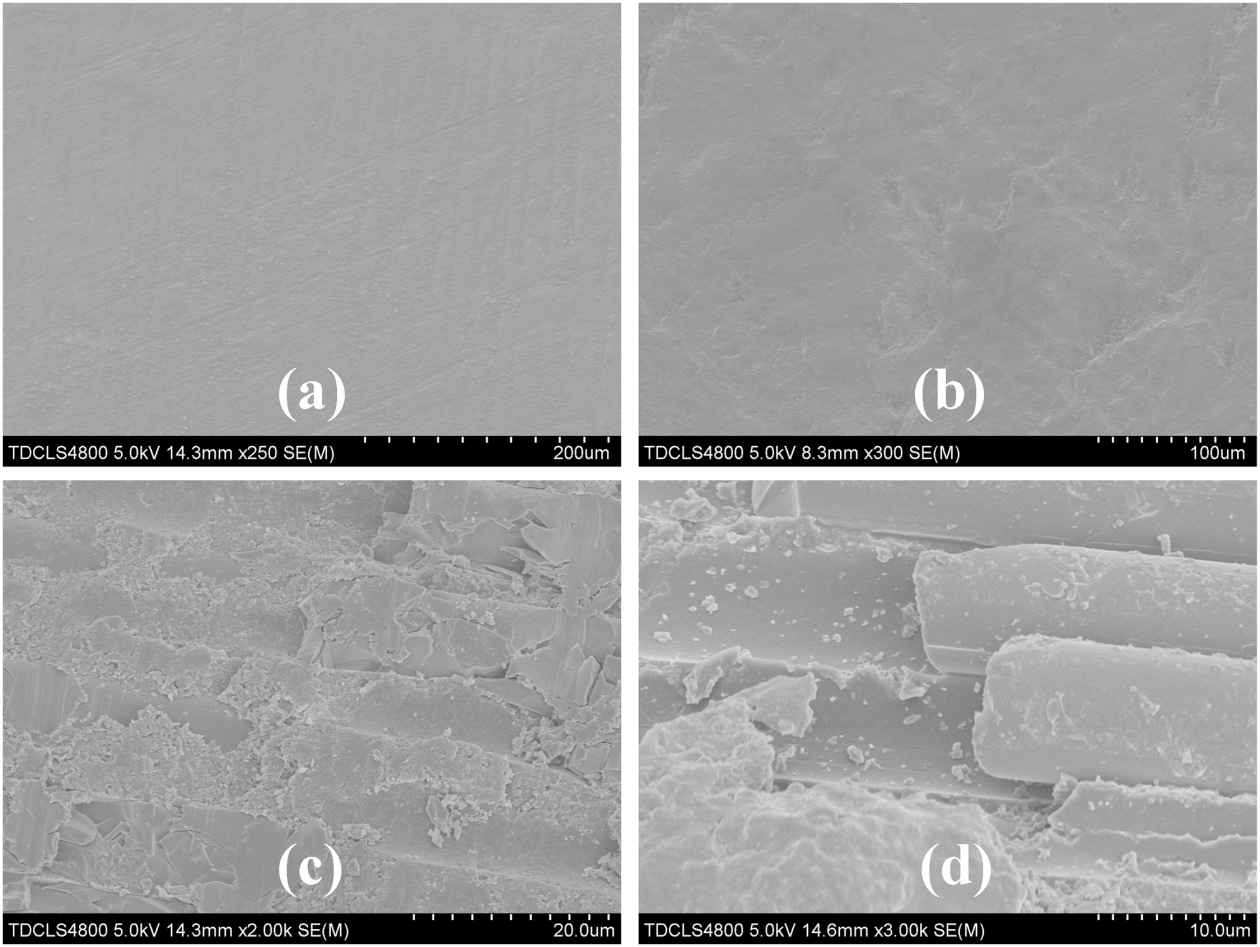
SEM images of the surface of the zirconia posts (a) before and (b) after sandblasting, the surface of the fiber posts (c) before and (d) after sandblasting.

Fig 3 shows the surface morphologies of the zirconia (Z-2) and fiber posts (F-2) (a, c) before and (b, d) after sandblasting. Both the zirconia and fiber posts showed smooth surfaces before sandblasting. After sandblasting, the zirconia post showed a rough surface due to surface damage. This damage was more serious for the fiber post. Fig 3d shows that a large area of polymer was removed with the breakage of the post, which decreased the strength of the fiber post.

**Fig 4:**
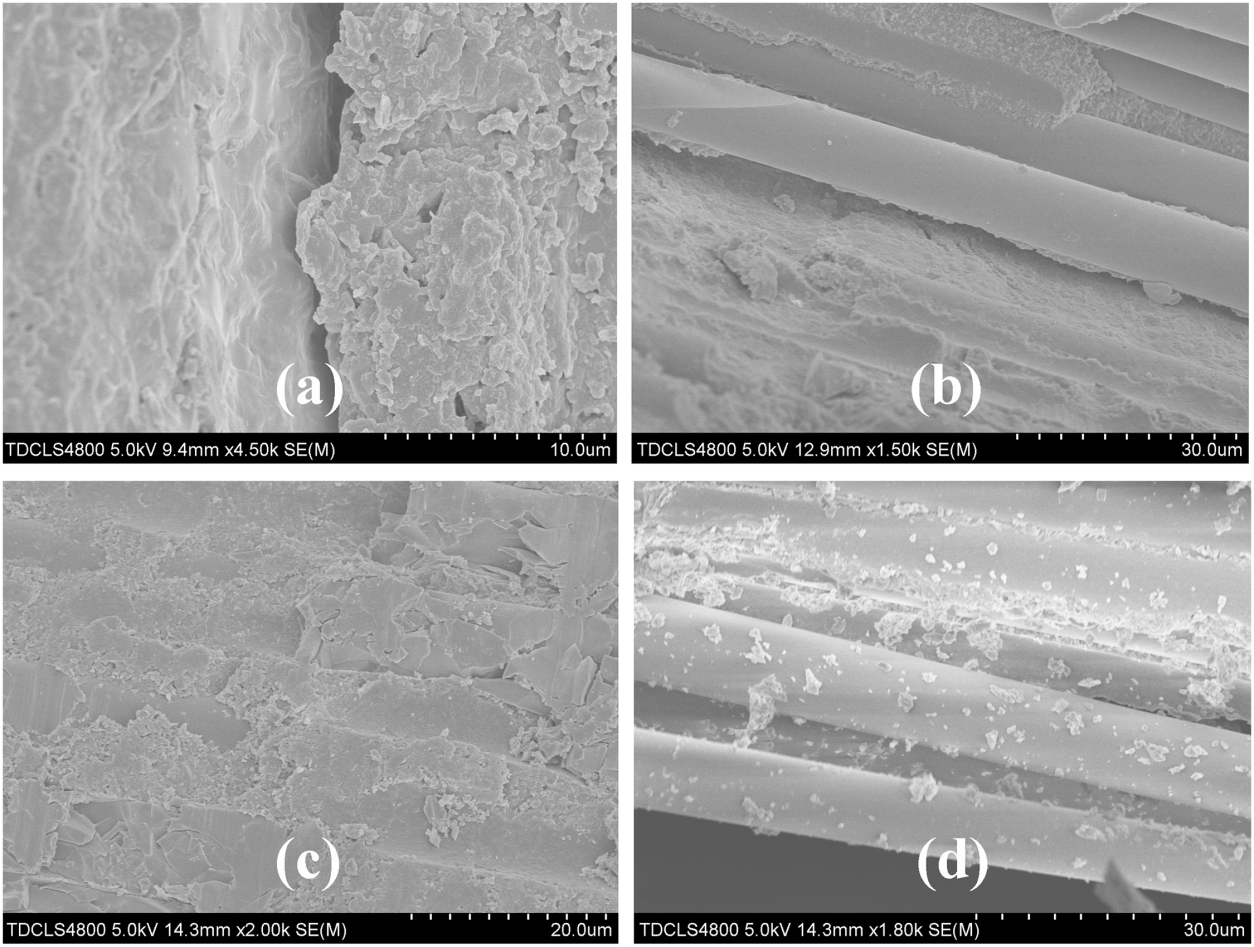
SEM images of the adhesive interface after push-out test. (a) A: zirconia post, B: resin cement. (b) A: fiber post, B: resin cement.

Fig 4 shows SEM images of adhesive interface between the post ((a) F-6 fiber and (b) Z-6 zirconia) and resin cement after thermocycling. The post and resin were separated at the adhesive interface by a gap because of thermal stress. Furthermore, large gaps were also observed at the interface between the zirconia post and resin cement. The remaining resin on the surface of the zirconia post indicated a good bonding strength between the zirconia post and resin cement based on the rough surface.

### Bonding strength

The bonding strengths between the fiber and zirconia posts and resin cement before and after thermocycling are shown in Table 3. It can be seen that the strengths of all of the specimens decreased to some degree after thermocycling. Two treatment methods of sandblasting and silanation were utilized to evaluate the effect on the bonding strength between the fiber or zirconia post and resin cement.

**Table 3:** Bonding strength between the post and the resin cement before and after thermo-cycling.

Group	Before Thermo-cycling	After Thermo-cycling
F-1	14.5 (3.2)^1^_a_	4.6 (2.0)^2^_a_
F-2	15.3 (2.8)^1^_a_	5.2 (2.1)^2^_a_
F-3	19.6 (4.2)^1^	10.7 (2.3)^2^_b_
F-4	22.2 (3.2)^1^	13.9 (2.9)^2^_c_
F-5	15.7 (3.5)^1^_a_	11.6 (2.9)^2^_b_
F-6	16.5 (3.3)^1^_a_	12.1 (2.7)^2^_bc_
Z-1	10.7 (2.1)^1^_a_	3.7 (1.2)^2^_a_
Z-2	17.4 (2.9)^1^_b_	11.8 (2.0)^2^_b_
Z-3	12.4 (2.5)^1^_a_	3.9 (1.2)^2^_a_
Z-4	16.1 (3.4)^1^_b_	6.5 (2.0)^2^_c_
Z-5	21.2 (3.7)^1^_c_	14.1 (3.1)^2^_d_
Z-6	24.5 (3.1)^1^_d_	17.1 (3.6)^2^_e_

(): SD. For each horizontal row in the mean value of the bond strength: superscript values with different numbers indicate statistically significant difference (P<0.05). For each vertical column in the mean value of the bond strength: subscript characters with same letters (a, b, c, d, e) indicate no statistically significant difference (P>0.05). The sample size for each experimental group was 15.

#### Sandblasting treatment

It can be seen that sandblasting improved the bonding strength (from 3.7 for Z-1 to 11.8 MPa for Z-2) between the zirconia post and cement because of the removal of a contamination layer and the rough surface that provided a large bonding area. In contrast, no significant increase in the bonding strength was observed for the fiber post. This result might be because of the damaged and broken post during sandblasting [35,36] with the resin removed from the surface.

#### Silane coupling

Despite no effect of sandblasting, the fiber post showed a dramatic increase in bonding strength with the silane coupling surface treatment. Compared with F-1, F-3 and F-4 showed high bonding strengths of 10.7 and 13.9 MPa, respectively, after thermocycling. This increased strength was attributed to the formation of covalent bonds (-Si-O-Si-) at the fiber/resin interface [37]. In addition, the two-bottle experimental primer showed a better strength for the fiber post than the one-bottle primer. This feature was attributed to the formation of a flexible silane layer at the silane/resin interface based on the two silicone-functional groups of BTS. BTS to some degree decreases the cross-linking density of the vinyl group of the methacryloxy portion of the γ-MPTS adsorbed onto the post surface [15]. Thus, the co-polymerization of BTS forms an elastic silane bi-layer that absorbs thermal stress during thermocycling. As a result, the bonding strength between the fiber post and resin was improved by chemical bonding at the silane/resin interface. However, this silanization effect was not observed for the zirconia post because no bonds formed at the interface, which was limited by the chemical structure of zirconia.

#### Combined effect of sandblasting and silane coupling

Because of the different effects for the fiber and zirconia posts, the combined treatment of sandblasting and silanization was utilized to improve the bonding strength. It can be seen that compared with F-3 (10.7 MPa) and F-4 (13.9 MPa) with the treatment of silane agents, sandblasting slightly decreased the bonding strength (F-5: 11.6 MPa and F-6: 12.1 MPa) between the fiber post and resin cement because of the damage and breakage of the post during airborne-particle abrasion. In contrast, the bonding strength between the zirconia post and resin cement dramatically increased after the combined treatment of sandblasting and silanization. Z-6 exhibited the highest bonding strength of up to 17.1 MPa after thermocycling, which was four times higher than Z-1 (3.7 MPa). This feature might be attributed to cross-linking at the interface because some alumina particles on the surface of the zirconia post generated during sandblasting reacted with the silane agent (γ-MPTS) to form chemical bonds. An organic layer anchored on the post led to the increase in bonding strength. This effect was also enhanced by using the two-bottle experimental primer, which formed a flexible bi-layer due to BTS with a decreased cross-linking density.

### Fracture mode

The types of fracture modes of the post before and after thermocycling after the push-out test are shown in Table 4. The fracture mode could be controlled by different surface treatment, which resulted in different bonding strengths between the post and resin cement. Without any treatment, the fiber (F-1) and zirconia (Z-1) posts usually showed interfacial fracture after the push-out test due to a relatively low bonding strength. As discussed above, sandblasting and silanization led to different bonding strengths between the post and resin cement, which was also confirmed by the different types of fracture modes. Generally, the treatment caused a change in the fracture mode from interfacial or mixed failure to cohesive failure. Specifically, despite no change after sandblasting, the fiber posts (F-2, F-3, F-4 and F-5) mainly showed cohesive or mixed failure after the treatment by silane agents because of the increased bonding strength. In contrast, the zirconia posts (Z-2 and Z-6) mainly showed cohesive or mixed failure after sandblasting due to the high bonding strength based on the rough surface, although the silanization effect showed no change in the fracture mode compared with Z-1. Importantly, the zirconia post (Z-5) treated by the silane agent without BTS also showed interfacial failure because of few chemical bonds at the interface, which was consistent with the bonding strength. The results indicated that the change from interfacial to cohesive or mixed failure mainly arose from high bonding strength between the post and resin cement controlled by sandblasting, silanization or a combined effect.

**Table 4:** The type of fracture mode of the post before and after thermo-cycling after the push-out test.

Group	Before Thermo-cycling [CMI]	After Thermo-cycling [CMI]
F-1	[7/5/3]^1^_a_	[0/5/10]^2^_a_
F-2	[8/5/2]^1^_ab_	[0/8/7]^2^_ab_
F-3	[13/2/0]^1^_acd_	[5/4/6]^2^_ac_
F-4	[15/0/0]^1^_cefg_	[8/4/3]^2^_c_
F-5	[13/1/1]^1^_ae_	[7/5/3]^1^_c_
F-6	[14/1/0]^1^_bdfg_	[6/5/4]^2^_bc_
Z-1	[3/2/10]^1^_a_	[0/0/15]^1^_a_
Z-2	[13/1/1]^1^_bc_	[6/6/3]^1^_b_
Z-3	[7/5/3]^1^_ab_	[0/0/15]^2^_a_
Z-4	[9/4/2]^1^_bc_	[0/3/12]^2^_a_
Z-5	[15/0/0]^1^_c_	[7/6/2]^2^_b_
Z-6	[15/0/0]^1^_c_	[13/2/0]^1^_b_

[C/M/I]: C) cohesive failure, M) mixed failure consisting of interfacial failure and cohesive failure, and I) interfacial failure. For each horizontal row in the type of fracture mode: superscript values with different numbers indicate a statistically significant difference (P<0.05). For each vertical column in the type of fracture mode: subscript characters with same letters (a, b, c, d, e, f, g) indicate no statistically significant difference (P>0.05), Complex chi-square 2) test. The sample size for each experimental group was 15.

## Conclusion

Within the limitations of the experimental procedures, the following could be concluded. We investigated the bonding strength between zirconia or fiber posts and resin cement using a push-out test after treatment by sandblasting, silanization or a combined method. The zirconia and fiber posts exhibit different bonding strengths and fracture modes after sandblasting and/or silanization. The SEM images showed that sandblasting led to the damage and breakage of the fiber post after thermocycling, whereas the zirconia post only showed a rough surface at the same treatment. A high bonding strength of up to 17.1 MPa between the zirconia post and resin cement was obtained after a combined treatment of sandblasting and silanization because of the rough surface and covalent bonds at the interface. This effect was enhanced by using γ-MPTS and BTS to form a flexible layer at the interface, which absorbed thermal stress to lower the loading. In contrast, the fiber post exhibited a high bonding strength of 13.9 MPa when only treated by silane agents because sandblasting resulted in damage of the fiber post. The results indicated that the improvement of the bonding strength between the post and resin cement could be controlled by different treatments based on covalent bonding and the surface roughness. A zirconia post with high bonding strength could potentially be used for the restoration of teeth in the future.
